# Combatting Persister Cells With Substituted Indoles

**DOI:** 10.3389/fmicb.2020.01565

**Published:** 2020-07-07

**Authors:** Sooyeon Song, Thomas K. Wood

**Affiliations:** ^1^Department of Animal Science, Jeonbuk National University, Jeonju, South Korea; ^2^Department of Chemical Engineering, Pennsylvania State University, University Park, PA, United States

**Keywords:** persisters, indole, substituted indole, resuscitation, formation

## Abstract

Given that a subpopulation of most bacterial cells becomes dormant due to stress, and that the resting cells of pathogens can revive and reconstitute infections, it is imperative to find methods to treat dormant cells to eradicate infections. The dormant bacteria that are not spores or cysts are known as persister cells. Remarkably, in contrast to the original report that incorrectly indicated indole increases persistence, a large number of indole-related compounds have been found in the last few years that kill persister cells. Hence, in this review, along with a summary of recent results related to persister cell formation and resuscitation, we focus on the ability of indole and substituted indoles to combat the persister cells of both pathogens and non-pathogens.

## Persister Cells

Persisters are stress tolerant cells that arise due to metabolic inactivity (Hobby et al., 1942; Bigger, 1944; Kwan et al., 2013; Pontes and Groisman, 2019) and without genetic change (Lewis, 2010). This dormancy was established by the original work with persisters showing non-growing *Staphylococcus aureus* cells are tolerant to penicillin (Hobby et al., 1942; Bigger, 1944). In contrast to *persistence*, which occurs in a small sub-population of cells, *resistance* occurs when mutations arise that allow growth in the presence of the antibiotic, and *tolerance* occurs when slow growth (e.g., stationary-phase cells) makes the entire population less susceptible to the antibiotic (Kaldalu et al., 2016; Kudrin et al., 2017). We have tried to clarify these terms to reduce the confusion in the persister-related literature (Wood et al., 2013; Kim and Wood, 2016, 2017; Kim et al., 2018a; Wood and Song, 2020) and tried to indicate how mistakes are being made in the persister literature by not waiting for a true plateau in the classic graph of the remaining viable cells during stress conditions that indicates the presence of persister cells (i.e., “biphasic” cell graph) (Song and Wood, 2020a). In addition, there is another term for the dormant state, “viable but non-culturable,” but we have demonstrated that the viable fraction of these cells is the same as persisters cells, at least for *Escherichia coli* and enterohemorrhagic *E. coli* (EHEC; Kim et al., 2018a).

Persisters have been shown to form from nutrient, antibiotic, acid, and oxidative stress (Hong et al., 2012; Kim et al., 2018a). Since nearly all cells starve (Song and Wood, 2018), persistence is likely a universal resting state of Bacteria and Archaea (Song and Wood, 2020b). Although persistence occurs to a small extent spontaneously (Balaban et al., 2004), it primarily arises as a highly regulated response to the environment (Dörr et al., 2010; Möker et al., 2010; Vega et al., 2012; Kwan et al., 2013, 2015; Hu et al., 2015; Song and Wood, 2020a; Wood and Song, 2020). This environmental response results in a small sub-population of stress-tolerant cells (∼1% or less) in biofilms and in stationary-phase cultures (Lewis, 2007, 2008).

As expected from a universal trait, persistence has been seen in all bacterial species tested (Van den Bergh et al., 2017). Strikingly, chronic infections are probably caused by resuscitated persister cells (Lewis, 2010; Van den Bergh et al., 2017); hence, they are important for cystic fibrosis (Lewis, 2007) and tuberculosis (Jayaraman, 2008). Therefore, understanding persistence is vital for developing more effective treatments for bacterial infections.

## Persister Cell Formation and Resuscitation

ppGpp has been linked to persistence (Korch et al., 2003; Nguyen et al., 2011; Chowdhury et al., 2016a; Svenningsen et al., 2019); hence, there is near consensus (Korch et al., 2003; Nguyen et al., 2011; Chowdhury et al., 2016a) for a role of the alarmone ppGpp for forming persisters (Svenningsen et al., 2019). However, until recently, the mechanism by which ppGpp leads to the formation of persister cells has been enigmatic.

To understand the link between ppGpp and persistence, it is informative to understand how ppGpp slows metabolism. To weather stressful conditions, cells reduce replication, transcription, and translation by synthesizing guanosine tetraphosphate and guanosine pentaphosphate (henceforth, ppGpp) (Gaca et al., 2015). ppGpp slows DNA replication by inhibiting DNA primase (Gaca et al., 2015), and ppGpp slows transcription by stimulating RpoS (sigma^S^, the stress response sigma factor for the stationary phase) and RpoE (sigma^E^, the stress response sigma factor for misfolded proteins in the periplasm) (Dalebroux and Swanson, 2012). ppGpp also inhibits the synthesis of purine nucleotides (Wang et al., 2019) and regulates purine homeostasis through its activation of nucleosidase PpnN (Zhang Y. E. et al., 2019). ppGpp slows translation by reducing the production of ribosomes (Shimada et al., 2013b).

**FIGURE 1:**
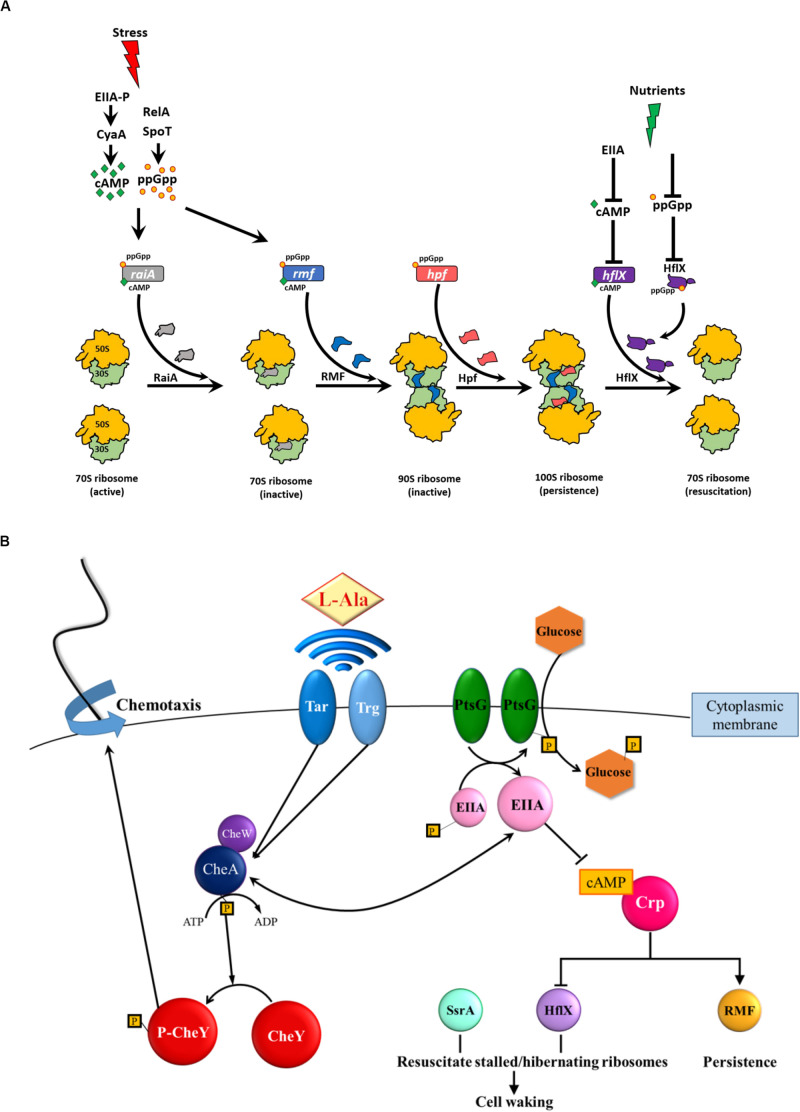
**(A)** ppGpp ribosome dimerization persister (PRDP) model for generating and resuscitating persister cells (Wood and Song, 2020). Myriad stresses (e.g., antibiotics, nutrient limitation, osmotic stress, and acid stress) induce the stringent response which results in (p)ppGpp (henceforth ppGpp) formation by RelA/SpoT in *E. coli* and generation of cAMP (e.g., upon glucose depletion via the phosphorylated glucose phosphotransfer enzyme, EIIA-P). ppGpp induces the genes encoding ribosome inactivation proteins, *raiA*, *hpf*, and *hpf* and cAMP induces *raiA* and *rmf*. RaiA inactivates 70S ribosomes, RMF converts 70S ribosomes into (inactive 90S ribosomes, and Hpf converts inactive 90S ribosomes into 100S ribosomes. At the protein level, ppGpp binds GTPase HflX to likely inactivate it (by blocking GTP binding), and cAMP represses *hflX*. With removal of the stress and the addition of nutrients, cAMP levels decrease (due to unphosphorylated EIIA) which stimulates HflX production; HflX dissociates 100S ribosomes into active 70S ribosomes and growth resumes. Used with permission. **(B)** Schematic of persister cell waking via alanine and glucose (Yamasaki et al., 2020). For alanine resuscitation, methyl-accepting chemotaxis proteins Tar and Trg sense the amino acid and relay this to chemotaxis response regulators CheA and CheY, which stimulate chemotaxis. For glucose resuscitation, phosphotransferase protein PtsG imports the sugar, which results in dephosphorylation of EIIA, reduction in cAMP, activation of chemotaxis, and ribosome rescue via HflX and SsrA. Spheres indicate proteins, diamonds indicate amino acids, hexagons indicate glucose, boxed P indicates phosphate, → indicates induction, and -∣ indicates repression. Used with permission from Elsevier (license #4807600114542).)

The activity of specific proteins is also reduced directly by ppGpp; for example, ppGpp binds and inhibits GTPases (Gaca et al., 2015). ppGpp also binds to GTPase HflX, the protein that activates dormant 100S ribosomes (Zhang et al., 2018), to prevent reactivation of inactivated ribosomes (Corrigan et al., 2016; Zhang et al., 2018) (Figure 1A). In addition, ppGpp inhibits the ribosome-associated GTPase Era that is involved in the biogenesis of 30S ribosome subunits (Wood et al., 2019).

Critically, for persister cell formation, ppGpp inactivates ribosomes (Figure 1A) by (i) inducing *rmf* (Izutsu et al., 2001), which encodes the ribosome modulation factor (RMF) that inactivates 70S ribosomes, (ii) inducing *hpf* (Prossliner et al., 2018), which encodes the hibernation promoting factor (Hpf), and (iii) inducing *raiA* (Prossliner et al., 2018), which encodes the ribosome-associated inhibitor (RaiA).

Others have focused on determining how ppGpp activates toxins of toxin/antitoxin (TA) systems and leads to persistence, but these works have been retracted (Maisonneuve et al., 2018a, b; Maisonneuve et al., 2019). Instead, we have proposed the simpler ribosome dimerization persister (PRDP) model (Figure 1A) in which ppGpp generates persister cells directly; i.e., without TA systems, by inactivating ribosomes by converting 70S ribosomes into inactive 100S ribosomes (Song and Wood, 2020b; Wood and Song, 2020). In support of this model, we found (Song and Wood, 2020b) that (i) most ribosomes in persister cells are inactive as 100S ribosomes, (ii) inactivation of RMF, Hpf, and RaiA leads to the formation of fewer persister cells and increases single-cell persister resuscitation substantially, and (iii) single-cell persister resuscitation is not affected by ppGpp levels. This model does not rely on TA systems for persister cell formation as their link to persistence is unconvincing (Conlon, 2016; Goormaghtigh et al., 2018; Pontes and Groisman, 2019).

Since persistence occurs without ppGpp, although at much lower levels (Chowdhury et al., 2016a), the PRDP model also includes a role for cAMP in activating RMF and Hpf without ppGpp, which leads to the formation of inactive and 100S ribosomes (Figure 1A). Specifically, starvation (e.g., glucose depletion) leads to elevated cAMP, induces *rmf* (Shimada et al., 2013b) and induces *raiA* (Prossliner et al., 2018). In addition, cAMP represses *hflX* (Lin et al., 2011). Therefore, cAMP plays a similar role to ppGpp for persister cell formation, since increased concentrations of both cell signals lead to ribosome inactivation and persistence in a sub-population of cells.

For persister cell resuscitation (Figure 1B), using single cells, we were the first to demonstrate persister cells resuscitate in an heterogeneous manner as they recognize external nutrients; the rate of resuscitation depends on the number of active ribosomes (Kim et al., 2018b). This heterogeneous nature of persister cell resuscitation was subsequently verified by others (Goormaghtigh and Van Melderen, 2019; Pu et al., 2019). Using single cells and searches over all *E. coli* proteins, we determined that persister cell resuscitation is initiated by recognizing external nutrients through receptors for chemotaxis (for amino acids) and phosphotransferase membrane proteins (for glucose) and does not require proteins specialized for persistence (Figure 1B) (Yamasaki et al., 2020). Resuscitation is also not primarily spontaneous but instead is based on the recognition of nutrients (Yamasaki et al., 2020). The presence of external nutrients (i.e., signals) is propagated to the cytosol by reducing concentrations of the secondary messenger cAMP; reduction in cAMP allows ribosomes stalled on mRNA to be rescued and inactive 100S ribosomes to be activated by HflX (Figure 1B) (Yamasaki et al., 2020). The resuscitating cells also initiate chemotaxis toward fresh nutrients, which is logical since nutrient depletion triggered persistence in the first place (Yamasaki et al., 2020). Therefore, we discovered specific signals for resuscitation, how those signals are detected by the exterior of the cell, how that external signal is propagated inside the cell via a second messenger, and that the cell initiates chemotaxis to nutrients upon waking (Yamasaki et al., 2020).

The PRDP model (Figure 1A) suggests that persister cell formation is an elegantly regulated response to stress. Experimental support for this idea is that spontaneous persisters are rare (Balaban et al., 2004) but various environmental forms of stress (e.g., antibiotics, hydrogen peroxide, acid) can convert almost the whole exponentially growing population into persister cells (Hong et al., 2012; Kwan et al., 2013). Similarly, the PRDP model suggests persister cell resuscitation is also an elegant environmental response rather than a spontaneous event, and our data with resuscitation with the amino acid alanine supports this (Yamasaki et al., 2020). Since nearly all cells face nutrient limitations and need dormant states to weather this stress, it is reasonable that cells require elegant regulation for both persister cell formation and resuscitation. Critically, the PRDP model suggests the “phenotypic switch” for persistence is predicated on the number of ribosomes inactivated; hence, only a small sub-population of stressed cells become persistent since they are the cells with a threshold level of ribosomes inactivated (Song and Wood, 2020a; Wood and Song, 2020); i.e., not all stationary cells are persisters since not all of these cells have a large enough percentage of ribosomes inactivated.

The PRDP model is general in that it is applicable to how persister cells form from various stresses since RMF has been shown to increase persistence dramatically in *E. coli* for myriad stresses including (i) ampicillin (Song and Wood, 2020b), ciprofloxacin (Song and Wood, 2020b), netilmicin (Tkachenko et al., 2017), gentamicin (McKay and Portnoy, 2015), acid (El-Sharoud and Niven, 2007), osmotic stress (Shcherbakova et al., 2015), and nutrient limitation (Yamagishi et al., 1993; Bubunenko et al., 2007). Furthermore, since RMF (Prossliner et al., 2018) and HflX (Basu and Yap, 2017) are conserved in bacteria, and Hpf is distributed in several kingdoms (i.e., prokaryotes and plants) (Akiyama et al., 2018), the PRDP model is probably applicable for the formation of the persister cells of many species. For example, persister cell formation of the opportunistic pathogen *Pseudomonas aeruginosa* also requires ppGpp (Nguyen et al., 2011) and both Hpf and ppGpp (but not RMF) are necessary for protecting ribosomes and ensuring the long term survival of *P. aeruginosa* during nutrient limitation (Akiyama et al., 2017). Furthermore, ppGpp plays a role in *hpf* expression in *P. aeruginosa* (Akiyama et al., 2018). Critically, for cysts of *Rhodospirillum centenum*, the first genes activated for waking encode for ribosomes and translation machinery (initiation, elongation, and release factors) (Ashok and Bauer, 2020); hence, it appears the PRDP model holds for many species and resting states.

## Indole Signaling

Indole, a product of tryptophan metabolism, is a multi-tiered signal in that it is an intra-species, inter-species, and interkingdom signal. As an intra-species signal, indole controls the quorum-sensing of *E. coli* (Lee et al., 2007a) primarily at low temperatures (Lee et al., 2008). As an interspecies signal, indole reduces the virulence of *P. aeruginosa*, which does not synthesize it, by reducing the virulence factors pyocyanin, rhamnolipid, 2-heptyl-3-hydroxy-4(1*H*)-quinolone, and pyoverdine (Lee et al., 2009a); this leads to increased competitiveness of commensal *E. coli* with *P. aeruginosa* (Chu et al., 2012). Also as an interspecies signal, indole reduces the virulence of EHEC by repelling it (negative chemotaxis), and by reducing its biofilm formation, motility, and attachment to HeLa cells (Bansal et al., 2007). Hence, we have suggested indole may be used as an anti-virulence compound (Lee et al., 2009a, 2015), and, indeed, indole was used successfully to reduce the virulence of *P. aeruginosa* in guinea pigs by reducing pulmonary colonization and increasing clearance in the lungs (Lee et al., 2009a). Twelve years later, the Sperandio group confirmed that indole reduces EHEC virulence in the gastrointestinal (GI) tract (Kumar and Sperandio, 2019). Furthermore, indole reduces the pathogenicity of *S. aureus* (Lee et al., 2013).

Strikingly, indole is an interkingdom signal, too. In the GI tract, indole produced by commensal bacteria tightens human epithelial cell junctions which reduces invasion by pathogens (Bansal et al., 2010; Shimada et al., 2013a). Also in the GI tract, we hypothesized that indole is probably hydroxylated by oxygenases to become an even more potent signal; for example, 7-hydroxyindole diminishes the virulence of *P. aeruginosa* more effectively than indole (Lee et al., 2007a). Furthermore, since many human and plant hormones are indole derivatives (e.g., indole-3-acetic acid, serotonin, melatonin, epinephrine), indole may be the archetype for cell hormones (Lee et al., 2007b). Further evidence showing indole in an interkingdom signal includes that for some plants (e.g., maize), indole is emitted to warn other plants of herbivores like the beet armyworm (Frey et al., 2000; Erb et al., 2015).

Moreover, indole reduces *E. coli* biofilm formation (Domka et al., 2006, 2007; Lee et al., 2007a, b, 2009b) and its production is reduced in biofilms (Domka et al., 2007). Also, by investigating the TA system YafQ/DinJ (Hu et al., 2015) and the phosphodiesterase DosP (Kwan et al., 2015), it was discovered that indole reduces *E. coli* persister cell formation.

## Conflicting Persistence Results With Indole Due to Diluents

Although there is one report claiming indole increases persistence with *E. coli* (Vega et al., 2012), consistent and overwhelming evidence has shown indole and substituted indoles *reduce* persistence in both Bacteria and Archaea (Hu et al., 2015; Kwan et al., 2015; Lee et al., 2016; Megaw and Gilmore, 2017; Li et al., 2019; Song et al., 2019; Manoharan et al., 2020; Masuda et al., 2020; Sun et al., 2020; Yam et al., 2020). For years, this was perplexing but it seems the most-probable reason for this different result lies in the solvent utilized to solubilize indole. Indole is relatively insoluble so to reach physiological concentrations (about 1 mM), a diluent must be used; dimethyl sulfoxide is the preferred solvent (Song et al., 2019) given it has little effect on cells if kept at less than 0.2 volume percent. In contrast, ethanol is not preferred due to its toxicity. Therefore, inconsistent results are most likely due to solvent effects. Hence, experiments with indole should include (i) suitable negative controls (i.e., solvent addition without indole) and (ii) multiple indole stock solutions to keep solvent addition uniform as indole concentrations are varied. In this way, indole is studied rather than the diluent.

## Indole-Related Compounds (Indigoids) Kill Persister Cells

**FIGURE 2:**
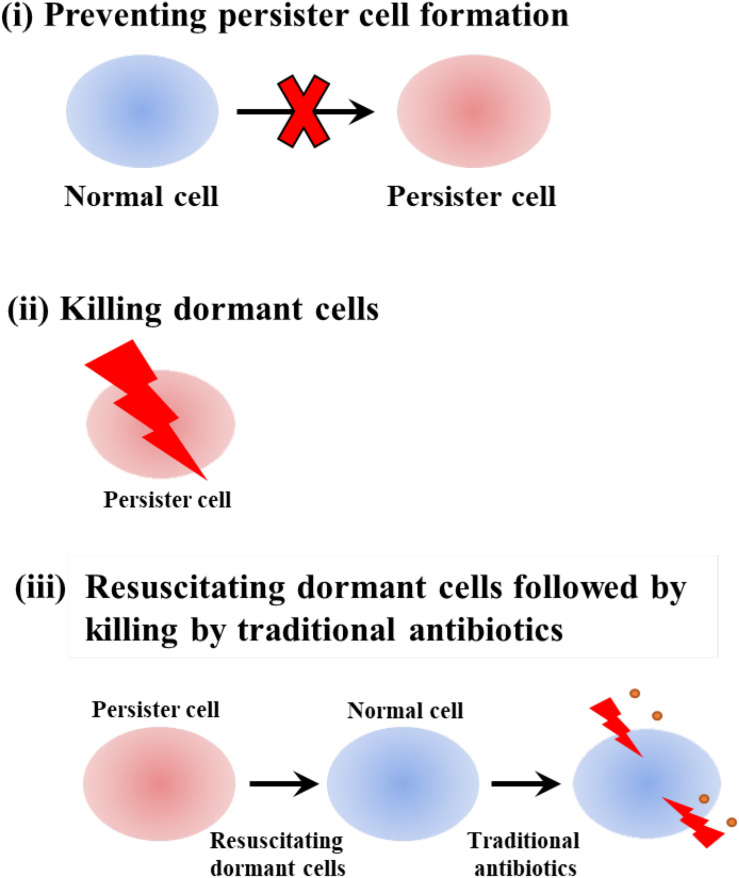
Schematic of combatting persister cells by **(i)** preventing persister cell formation, **(ii)** killing dormant cells, and **(iii)** resuscitating dormant cells followed by killing by traditional antibiotics.

We previously organized chemicals used to combat persister cells into three categories: (i) preventing persister cell formation, (ii) killing dormant cells, and (iii) resuscitating dormant cells followed by killing by traditional antibiotics (Figure 2) (Wood, 2016). As we show in this section, indigoids primarily inhibit persistence by killing dormant cells as a result of membrane damage.

For *E. coli*, we discovered 2 mM indole reduces persistence (Hu et al., 2015; Kwan et al., 2015) and found the effect with ampicillin to be about 52-fold. A corroboration of the reduction of persistence by indole with the same strain was published recently along with the interesting result that indole also reduces heat tolerance in *E. coli* (Masuda et al., 2020). The ability of indole to kill a wide range of persister cells is illustrated by its ability to also kill the persister cells of the archaeal strain *Haloferax volcanii* (up to 188-fold increase in killing) (Megaw and Gilmore, 2017).

**FIGURE 3:**
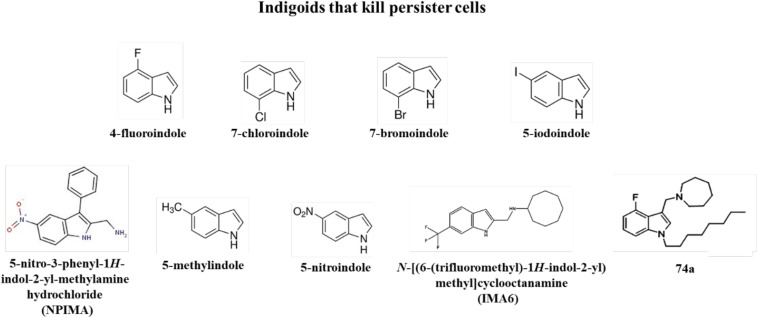
Indigoids that kill persister cells.

However, substituted indoles are even more active in killing persister cells. For example, by using our method to convert nearly the whole *E. coli* bacterial population into persister cells (Kwan et al., 2013; Kim et al., 2018b), so compounds may be more readily screened for persister killing, 36 indole derivatives were assayed for persister killing including halogenated-, methoxy-, methyl-, and nitro-indoles. From this screen, it was found that halogenated indoles such as 4-fluoroindole, 7-chloroindole, 7-bromoindole, and 5-iodoindole (Figure 3) eradicate *E. coli* persisters. Moreover, 5-iodoindole was the most effective indigoid with 1500-fold greater activity than unsubstituted indole with *E. coli* (Lee et al., 2016). 5-Iodoindole also eradicated *S. aureus* persister cells but was not effective with *P. aeruginosa* (Lee et al., 2016). Hence, a new class of powerful anti-persister compounds was discovered based on indole that eradicates both Gram negative and Gram positive cells.

Recently, a substituted indole was found that is effective in killing *P. aeruginosa* persister cells: 5-nitro-3-phenyl-1*H*-indol-2-yl-methylamine hydrochloride (NPIMA, Figure 3) (Song et al., 2019). NPIMA was discovered by converting the *E. coli* exponential cell population into persister cells by pre-treating with rifampicin to stop transcription (Kwan et al., 2013; Kim et al., 2018b), then performing the first, direct, high-throughput screening of persister cells (Song et al., 2019); a 10,000-member library of druglike compounds was utilized. It was found that NPIMA was more effective than 5-iodoindole (Lee et al., 2016) and cisplatin (Chowdhury et al., 2016b) in killing *E. coli* persisters. Importantly, NPIMA also eradicated both *P. aeruginosa* and *S. aureus* persisters. Critically, the mechanism of NPIMA persister killing was determined and found to be due to membrane damage (Song et al., 2019). Furthermore, *E. coli* resistance to NPIMA did not occur in a week, and NPIMA was found effective in a wound model with *P. aeruginosa* and *S. aureus* (Song et al., 2019).

Indole derivatives have also been combined both with antibiotics and metals to increase their effectiveness in persister cell killing. For example, 5-methylindole (Figure 3) combined with tobramycin kills methicillin-resistant *S. aureus* and Staphylococcus *epidermidis* persisters (Sun et al., 2020). In addition, 5-nitroindole (Figure 3) kills *E. coli*, *P. aeruginosa*, and *Enterobacter tabaci* persister cells, and its effectiveness was increased by combining it with copper and zinc nanoparticles (Manoharan et al., 2020).

Since tuberculosis kills 1.5 million people every year (Yang et al., 2017), it is imperative that compounds that eradicate persister cells related to mycobacteria be identified. Critically, a substituted indole, *N-*[(6-trifluoromethyl)-1*H*-indol-2-yl)methyl]cycloocctanamine (IMA6, Figure 3), has been identified that kills *Mycobacterium abscessus* persister cells (Yam et al., 2020). In addition, 4-fluoro and 6-methoxyindoles combined with a cationic amphiphilic motif (e.g., lipophilic n-octyl side chain at position 1 and a positively charged azepanyl or 1,4-dioxa-8-azaspiro[4.5]decane moiety at position 3, Figure 3 for compound 74a) have been identified that kill *Mycobacterium tuberculosis* and kill *Mycobacterium bovis* persister cells by damaging the membrane (Yang et al., 2017). No resistance was found to compound 74a in 8 weeks, and the compound was active on *S. aureus* but had no activity on *E. coli* (Yang et al., 2017). Hence, substituted indoles are effective against some of the most dangerous pathogens that are often in non-replicating states and require treatments for 1 year with current antibiotics.

## Indole Prevents Exit From Dormancy in Consortia

In addition to killing persister cells, indole also has another remarkable trait: it selectively allows *E. coli* cells to resuscitate from dormancy while preventing other cells from resuscitating (Zhang W. et al., 2019). Specifically, indole has no effect on *E. coli* resuscitation, but indole prevents *P. aeruginosa* persisters from waking (Zhang W. et al., 2019). Furthermore, indole allows *E. coli* to outcompete *P. aeruginosa* (Zhang W. et al., 2019). Critically, indole has no toxicity with non-dormant and dormant *P. aeruginosa* cells at physiological levels (Zhang W. et al., 2019) so the inhibition of resuscitation is not due to toxicity and not due to a difference in the number of *P. aeruginosa* persister cells that are formed. Unfortunately, the mechanism of indole inhibition has not been determined.

This indole phenotype likely gives *E. coli* a fitness advantage over its competitors and may be one of the main reasons indole is secreted from *E. coli* at such high levels, around 0.7 mM (Domka et al., 2006). These results are also physiologically relevant since both *E. coli* and *P. aeruginosa* are found together in the GI tract as *P. aeruginosa* is present in up to 12% of healthy individuals (Bodey et al., 1983) and is found sometimes in the GI tract of critically ill surgical patients (Marshall et al., 1993). Since indole from *E. coli* also reduces many of the quorum-sensing-related virulence factors of *P. aeruginosa* as an inter-species signal (Lee et al., 2009a), these new results (Zhang W. et al., 2019) indicate indole from *E. coli* both reduces *P. aeruginosa* virulence as well as prevents its resuscitation from the persister state.

## Perspectives

It seems the most important aspect of indole secretion by *E. coli* is not related to the control of its own gene expression as a quorum-sensing signal but instead lies in the influence of indole on its neighbors as an interkingdom and interspecies signal. For example, indole controls few genes (Wang et al., 2001; Lee et al., 2008); in contrast, indole is clearly beneficial to the host of *E. coli* (e.g., by tightening epithelial cell junctions to prevent sepsis) (Bansal et al., 2010) and indole is beneficial for controlling the competitors of commensal *E. coli* since indole both reduces the virulence (Lee et al., 2009a) and the resuscitation of the pathogen *P. aeruginosa* (Zhang W. et al., 2019) as well as reduces the virulence of EHEC (Bansal et al., 2007; Lee et al., 2007a).

Making use of our discovery that indole reduces persistence (Hu et al., 2015; Kwan et al., 2015), many labs now have independently identified indigoids that are potent for killing persister cells. Future work on the ability of these substituted indoles to enter host cells and kill intracellular persisters would be interesting; note that indole itself is actively transported in *E. coli* by Mtr but has some less-efficient diffusion into the bacterial cell (Vega et al., 2012). Hence, one can be sanguine about the future and bringing some of these compounds to market to treat recalcitrant infections.

## Author Contributions

Both authors contributed to the article and approved the submitted version. TW conceived the review. SS and TW authored the manuscript.

## Conflict of Interest

The authors declare that the research was conducted in the absence of any commercial or financial relationships that could be construed as a potential conflict of interest.
